# Auditory Nerve Spike Generator Modeled as a Variable Attenuator Based on a Saddle Node on Invariant Circle Bifurcation

**DOI:** 10.1371/journal.pone.0045326

**Published:** 2012-09-18

**Authors:** Mark Ospeck

**Affiliations:** Boulder, Colorado, United States of America; National Research & Technology Council, Argentina

## Abstract

Mammalian inner hair cells transduce the sound waves amplified by the cochlear amplifier (CA) into a graded neurotransmitter release that activates channels on auditory nerve fibers (ANF). These synaptic channels then charge its dendritic spike generator. While the outer hair cells of the CA employ positive feedback, poising on Andronov-Hopf type instabilities which make them extremely sensitive to faint sounds and make CA output strongly nonlinear, the ANF appears to be based on different principles and a different type of dynamical instability. Its spike generator “digitizes” CA output into trains of action potentials and behaves as a linear filter, rate-coding sound intensity across a wide dynamic range. Here we model the spike generator as a 3 dimensional version of a saddle node on invariant circle (SNIC) bifurcation. The generic 2d SNIC increases its spike rate as the square root of the input current above its spiking threshold. We add negative feedback in the form of a low voltage-threshold potassium conductance that slows down the generator’s rate of increase of its spike rate. A Poisson random source simulates an inner hair cell, outputting a series of noisy periodic current pulses to the model ANF whose spikes phase lock to these pulses and have a linear frequency to current relation with a wide dynamic range. Also, the spike generator compartment has a cholinergic feedback connection from the olive and experiments show that such feedback is able to alter the amount of H conductance inside the generator compartment. We show that an olive able to decrease H would be able to shift the spike generator’s dynamic range to higher sound intensities. In a quiet environment by increasing H the olive would be able to make spike trains similar to those caused by synaptic input.

## Introduction

Mammalian auditory nerve fibers (ANF) respond to faint sounds by increasing the frequency of their action potentials by a small amount, but they are also able to respond to a wide dynamic range of sound inputs by making large increases in their spike rate [1]. ANF are able to adjust to noisy environments, enabling them to more accurately rate-code loud tones [1]. When they use the transient frequency of their spike rate to encode the intensity of a tone, ANF function as linear filters [2]. But in general, spike generator output is known to be intrinsically nonlinear, with spiking typically turning on abruptly when an input current threshold is passed; its rate rising steeply with increasing input current [3]–[5]. The apparent contradiction of the ANF behaving as a sensitive but also wide range linear filter is referred to as the dynamic range problem in mammalian hearing [6]. Previously, negative feedback has been investigated as one likely means for slowing down a spike generator’s initial rate of increase (specifically in the case of cortex pyramidal neurons [7]). This result has been mathematically generalized. It is a generic property of strongly nonlinear spike generators that negative feedback is able to linearize their firing frequency as a function of input current (*f - I* curve), provided that their no feedback *f - I* curve is sufficiently nonlinear [8]. It is this same sort of negative feedback that linearizes an op amp’s output [9]. The ANF has an extremely wide output spiking range from 0 to about 300 Hz [1]. We will show that this along with other evidence argues that its spike generator is based on a modified version of dynamical instability called a saddle node on invariant circle (SNIC) bifurcation. We show that when the SNIC bifurcation adds a dynamical variable that provides fast voltage-dependent negative feedback, it becomes well-suited for linear rate-coding across a wide dynamic range.

Each inner hair cell (IHC) originates about 20 ANF. Most of these fibers are very sensitive, with their thresholds near 0 dB spl that correspond to sound waves which make only 20 micro Pascal pressure fluctuations [1]. At the same time the fiber’s dynamic range is about 40 dB, or about a hundred fold pressure range [1]. A single IHC activates excitatory AMPA channels at its synapse to an unmyelinated end segment of an ANF dendrite, which has a capacitance of only about 1 picoFarad and contains the nerve’s spike generator [10], [11]. This is different from a typical spike generator which would be located in a neuron’s soma and have to work against a much larger 10–30 pF capacitive load. However, most neurons have many synaptic inputs to their dendrites from other neurons, while the ANF synapses only with a single IHC. Thus the ANF is unusual for its 1–1 coupling to an IHC and for placing its high output spike generator into a small and leaky compartment. Note that by lowering its compartment capacitance, the generator’s capacitive admittance is minimized, thereby reducing capacitive shunting of currents associated with steep rising action potentials and excitatory post synaptic potentials (epsps). In addition, there is an efferent feedback connection from the lateral olive directly to the tiny generator compartment, and there is experimental evidence that cholinergic feedback via a second messenger pathway is able to turn off inward leak currents to the generator compartment [9]. We show how turning off inward leak shifts the generator’s dynamic range upwards so that louder tones are more accurately encoded into spike trains.

## Results

Below about 3 kHz, mammalian ANF phase lock their action potentials to the sound waves sensed by their respective IHC [12]. In mouse, fast sodium channels, the kind typically found in node of Ranvier spike repeaters, have been localized to the IHC-ANF post synapse dendritic segment just before myelination begins [11]. This leaky dendrite, with a diameter less than one micron, has been identified as the ANF spike generator. Experiments on rat dendrite also reveal the presence of both high and low voltage threshold potassium conductance, together with an H conductance that appears to be under control of the lateral olive [10]. H is a monovalent cation current that conducts mainly sodium into the cell. It is voltage sensitive, but unlike most other voltage-gated channels, H turns on with hyperpolarization (hence its name, “H”). Also, H gates very slowly with voltage changes, typically taking hundreds of milliseconds to seconds to open or close. Here since we are considering only short tone bursts (such as a formant frequency for a vowel sound which lasts for only several hundred milliseconds) H is approximated as a fixed inward leak. AMPA conductance with a reversal potential near 0 mV, and a linear current-voltage relation, is the main synaptic conductance in rat ANF [10]. A quantal epsp, or “mini” due to single IHC vesicle release is about 2.4 mV tall with a rise time of about 1 ms, and its ∼40 pA current charges the ∼1.3 pF capacitance (*C*) of the spike generator compartment [10]. Previously we used these experimental results to construct a 10 compartment biophysically-based model of the dendritic spike generator for a high frequency non-phase-locking ANF that employed a square wave input current [13]. The fast negative feedback that gave it a wide dynamic range was due to a second messenger pathway. A second 10 compartment model was driven by 440 Hz current pulses, with their size given by a Poisson random variable, and this one used low threshold voltage-gated potassium conductance to provide its fast negative feedback. In this paper we will focus on a reduced 3d version of this second multi compartment model in order to more simply and clearly explain how and why this type of spike generator is able to obtain a linear *f - I* curve with a wide dynamic range. For this reduced model, we use only that the dendrite has both high and low threshold voltage-gated potassium conductance, fast sodium conductance, inward leak H conductance, and that it is driven by a noisy but periodic stream of AMPA current pulses.

There are two fundamentally different types of dynamical instabilities that are able to fire neuronal action potentials; the Andronov-Hopf (AH) bifurcation and the saddle node (SN) bifurcation [4], [5]. To obtain a spike generator with a wide dynamic range, a specific type of saddle node bifurcation called a SNIC (saddle node on invariant circle) appears to be the best choice to start with, since its spike rate increases only as the square root of the current above the bifurcation. For other bifurcations, the rate of increase is faster. Above an SN bifurcation, the rate increases as the log of the current, and at an AH bifurcation, the rate discontinuously jumps from zero into a narrow frequency range [5]. The very wide spike frequency range of an ANF essentially rules out its being based on an AH bifurcation. The argument against an SN bifurcation would be that its spikes tend to be followed by after depolarizations, while ANF spikes are followed by after hyperpolarizations, the type of spikes produced by a SNIC bifurcation [5], [12]. However, there is still a steep initial rise in the spike rate above a SNIC bifurcation, and this eats up a lot of dynamic range. The idea will be to start with a 2d SNIC and then use fast voltage-dependent negative feedback to stretch its dynamic range.

**Figure 1:**
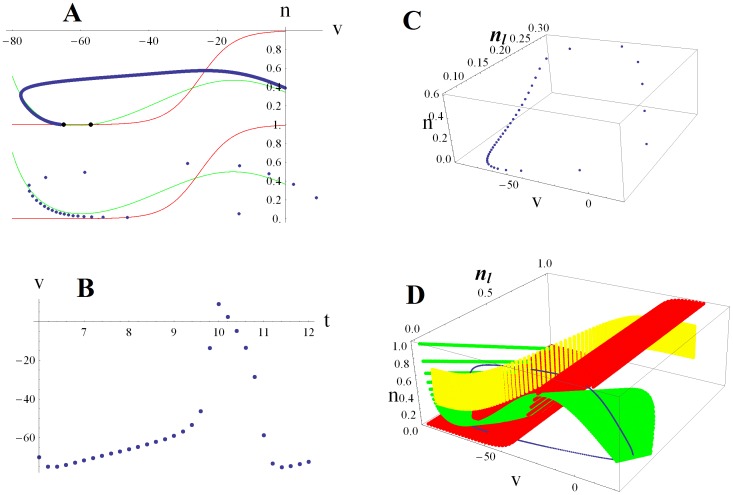
Trajectories for a 2d versus 3d saddle node on invariant circle (SNIC) bifurcation. A. Phase plane plots for a 2d SNIC that show the trajectory (blue) with a fast voltage variable ***v*** plotted against a slower ***n***, the high threshold voltage-gated potassium conductance open probability. ***dv/dt = 0*** (green) is the voltage nullcline; when the trajectory goes above it then ***dv/dt<0*** so that the voltage decreases. Left of the red curve ***dn/dt<0*** so that the open probability of the potassium conductance decreases. When input current is small the system is poised below the bifurcation so that the trajectory ends on the stable node (black dot upper plot). As input current increases the voltage nullcline is raised (green curve lower plot) until the stable node merges with the saddle node to its right and both disappear. The trajectory then starts orbiting in a series of action potentials (blue dots spaced at equal time intervals in the ***n*** vs. ***v*** plot. B. ***v*** vs. ***t*** plot showing the bottleneck where the spiking trajectory slows down (closely spaced blue dots in parts A and B where ***v*** increases relatively slowly). Further small increases in input current slightly raise the voltage nullcline, which has the effect of rapidly increasing the spike rate. C. 3d SNIC in which the rate of increase of spike rate is slowed by adding a slow low threshold voltage-gated potassium conductance to the generic 2d SNIC. When this conductance with gating variable ***n_l_*** is included, the spiking trajectory gets trapped for a longer time in the bottleneck, being mainly confined to the ***n*** *,* ***n_l_*** plane with little increase in the ***v*** direction. Slowdown in the rate of voltage increase is most easily seen by observing that the number of dots in the bottleneck approximately doubles as compared to part A (same input current and time intervals). D. Null surfaces corresponding to 3d SNIC: ***dv/dt = 0*** (green), ***dn/dt = 0*** (red) and ***dn_l_/dt = 0*** (yellow) show the turning points of this 3 dimensional trajectory (blue line). The slowed trajectory gets wrapped close behind the green null surface where ***dv/dt*** is positive, but small due to ***n_l_*** turning off slowly.

The canonical 2d form of a SNIC bifurcation pairs a fast voltage variable ***v*** with a slower high voltage threshold potassium current. In the FitzHugh-Nagumo, Morris-Lecar, or persistent sodium current models, a constant level of input current *I* is used as a bifurcation parameter that raises the voltage nullcline, the curve where ***dv/dt = 0*** [4]. When *I* is raised above the critical value *I_b_*, the result is a bifurcation that produces a train of action potentials–-such as when a saddle and node coalesce and annihilate (black dots in Fig. 1A top). After this, in the phase plane plot of the SNIC, there develops a bottleneck in the spiking trajectories due to their proximity to the “ghost” of the saddle and the node (Figs. 1A bottom and 1B). Larger *I* moves the trajectory farther away from the bottleneck, thus increasing the frequency of the action potentials **(** ***f ∼ (I - I_b_)^1/2^*** close above a SNIC [4], [5]). We use the fact that the ANF has low threshold voltage-gated potassium conductance to add a third dynamical variable ***n_l_*** (Eqn. 3) to the 2d SNIC’s voltage variable ***v*** (Eqn. 1) and high-threshold potassium conductance gating variable ***n*** (Eqn. 2). ***n_l_*** is a slower voltage gating variable on the low threshold potassium conductance, and adding it creates a 3d SNIC with a bottleneck in the ***n*** *,* ***n_l_*** plane (Fig. 1C). Null surfaces that correspond to ***dv/dt = 0*** (green), ***dn/dt = 0*** (red) and ***dn_l_/dt = 0*** (yellow) show the turning points of the trajectory and how it gets trapped close behind the green voltage null surface (Fig. 1D)
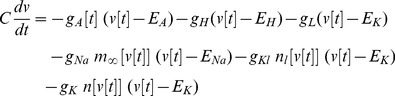
(1)

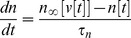
(2)

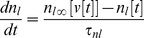
(3)


Six currents charge the capacitance *C* of the spike generator compartment (Eqn. 1). *g_H_* is the amount of inward leak H conductance, *E_H_* its reversal potential, *g_Na_* sodium conductance, *E_Na_* its reversal potential, *g_L_* outward leak potassium conductance, *g_Kl_* low threshold voltage-gated potassium conductance, *g_K_* high-threshold voltage-gated potassium conductance, and *E_K_* the potassium reversal potential. ***m_∞_[v[t]]***, ***n_∞_[v[t]]*** and ***n_l∞_[v[t]]*** give the voltage dependencies for the gating of the sodium conductance, high threshold potassium conductance and low threshold potassium conductance. For simplicity, the sodium conductance is assumed to be instantaneously fast and without its slower voltage inactivation via a blocking ball, as is done in the persistent sodium current model [5]. ***τ*** *_n_* and its 4 times slower counterpart ***τ*** *_nl_* are the time constants for the gating of the high and low threshold potassium conductances, respectively. ***g_A_[t]*** is the time-dependent AMPA conductance and *E_A_* its reversal potential. AMPA conductance is assumed to have an elementary conductance pulse of size *dg_A_*. The probability for a pulse of size *x dg_A_* is given by a Poisson distribution ***P[x]  =  Exp[-N] N^x^/x!*** with mean size *N*. This 3d SNIC model uses dimensionless units and its Mathematica notebook is given in the methods section.

**Figure 2:**
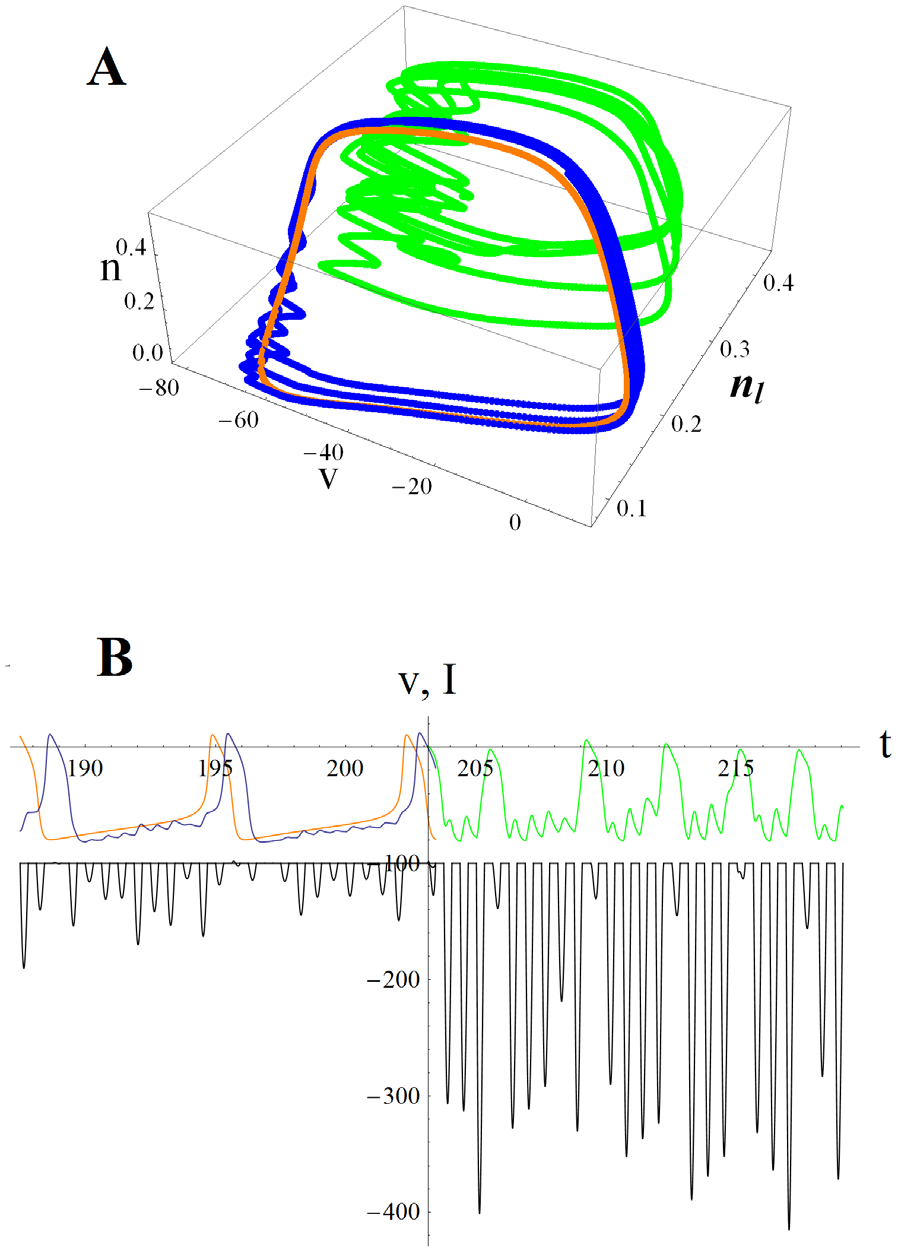
ANF spike generator modeled as a 3d version of a SNIC bifurcation. A. Spiking trajectories are shown for small (*N*  = 2 blue) and large (*N*  = 20 green) noisy periodic input currents. For comparison a trajectory driven only by increasing H conductance by 50% is shown (orange). ***v*** is membrane potential, ***n*** is the open probability of the high threshold voltage-gated potassium conductance (usually referred to as the delayed rectifier) and ***n_l_*** the open probability of the low threshold voltage-gated potassium conductance (negative feedback that slows the rate of spike rate increase). B. Spike trains for the same small input (blue) and large input (green) cases. Synaptic AMPA input currents (black) that correspond to an average of *N* vesicles released by the inner hair cell per sound wave cycle are given by a Poisson random variable. H current driven spike train is in orange. Experiments show that H can be altered by cholinergic feedback from the olive via a second messenger pathway [10]. Feedback control of H would be able to generate spike trains similar to those caused by synaptic input.

Trajectories are shown for small and large noisy periodic AMPA input currents (Fig. 2A). Note that for large input (*N* = 20 green), the open probability of the low threshold potassium conductance (amount of negative feedback) is approximately double that for small input (*N* = 2 blue). Spike trains corresponding to these two cases, and the sequences of noisy periodic current pulses driving them, are shown in Fig. 2B. The lateral olive sends a cholinergic efferent able to alter H conductance directly to the spike generator compartment [10]. We compare an AMPA current driven spike train (*N* = 2 blue) to one excited only by H current (orange; Fig. 2A, B). An H current under control of the olive would appear to be able to make spike trains like those driven by synaptic input. Here there would be no phase-locking to an external sound wave. Rather a frequency place-coded ANF would be responding as if there were acoustic power present at a particular frequency. For example, in a quiet environment a musician with a lateral olive that’s able to vary its feedback in the several Hz range might be able to “play” a series of musical tones in his head by raising and lowering his H conductance (being deaf like Beethoven might make this easier). This sort of effect would be an example of a generative model in which feedback to the sensory periphery should in a sense be able to reconstruct or generate sensory input.

**Figure 3:**
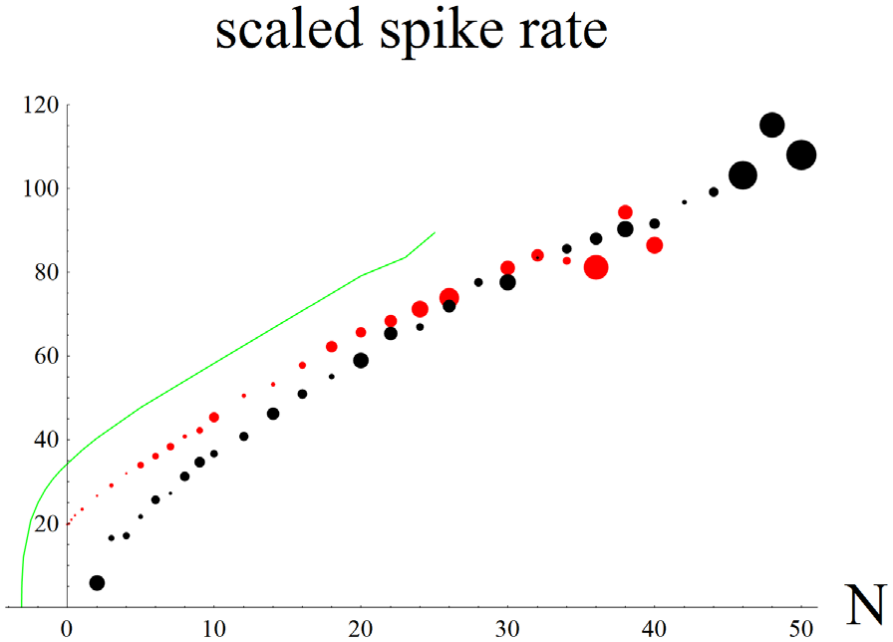
Spike rate vs. *N*, the average number of vesicles released by the IHC per sound wave cycle for the 3d SNIC model. The size of the data points shows the standard error in the spike rate, which increases with *N* (red; *gKl* = 8, *gL* = 5, *gH* = 1). The plot is similar to spike frequency vs. input current and is made linear across approximately a 48 dB range (from *N*∼1/8 to *N*∼32) by fast negative feedback from the low threshold potassium conductance *gKl*. For small input the small standard error allows the *N*  = 0, 1/8, 1/4 excitations to be resolved from each other, while for large inputs the accompanying large standard error limits the ability to resolve intensities above *N* = 32. Turning off inward leak H current moves dynamic range to higher sound intensities (black; *gKl* = 8, *gL* = 5, *gH* = 0; ∼27 dB range from *N* = 2 to 46). H was turned off as if it were under the control of cholinergic feedback from the olive. Here the standard error starts out large for the small spike rates just above the bifurcation (*N* = 2), decreases away from the bifurcation and then increases for large inputs. For comparison we show the loss of dynamic range and nonlinear rise of the spike rate due to turning off the low threshold potassium conductance (green; *gKl* = 0, *gL* = 5, *gH* = 1). This curve was extended to negative *N* to show the square root shape in the rise of the spike rate above the now 2d SNIC bifurcation. Fluctuations in the size of the standard errors occurred because spike rates and standard errors were calculated over short time intervals, trying to reproduce the approximately one quarter second that the auditory pathway has to resolve the intensities of specific formant frequencies in order to be able to identify a particular vowel sound in real time. At a given frequency that is sensed by a particular IHC small numbers of ANF rapidly and accurately determine the intensity of the tone, each using only a small number of spikes.

Fast voltage-dependent negative feedback from the low threshold potassium conductance linearizes the *f – N* curve across approximately a 48 dB wide dynamic range (red data points in Fig. 3). The standard error of the spike rate is evaluated as if for short tone bursts and it is important for estimating the dynamic range of the ANF. The size of each data point specifies its standard error for a short time series of spikes. For example, small standard error allows an *N* = 1/8 excitation to be resolved from *N* = 1/4, but large standard error muddles intensity discrimination above *N* = 32. Also shown is that if an efferent feedback from the olive were able to turn off H conductance then it would be able to adjust the ANF’s dynamic range upwards (black data points in Fig. 3). ANF dynamic range decreases to ∼27 dB, but intensity resolution is improved in the upper intensity range, which is consistent with experiments on auditory nerve fibers in noisy environments [1]. Upwards shifting the dynamic range increases the information content of spike trains that encode a higher range of sound intensities. Note that for accurate rate coding that it’s better to be at some distance away from the bifurcation. Near the bifurcation small spike rates become highly variable (large black data point at *N* = 2). Low threshold ANF may have high 20–100 Hz spontaneous spike rates [12] in order to be away from the bifurcation. It appears that some output dynamic range is sacrificed in order to improve the accuracy of fast rate coding of sound intensity. Also, for comparison, low threshold potassium conductance was turned off to obtain the green curve in Fig. 3 that shows the square root shape in the rate of spike rate increase above a 2d SNIC bifurcation.

**Figure 4:**
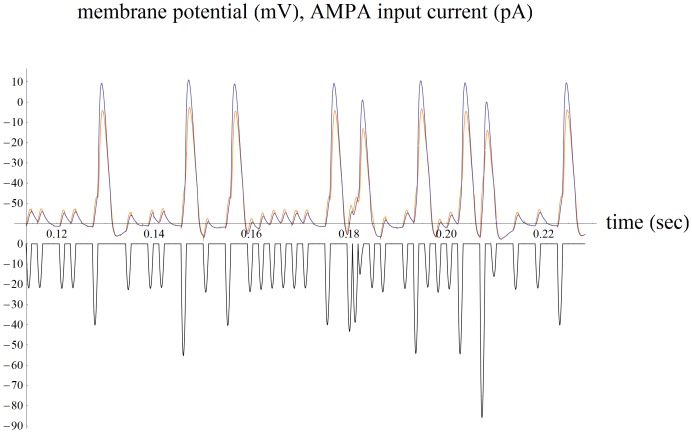
For comparison a spike train is shown from a biophysically-based 10 compartment model of the ANF dendritc spike generator. The simplified 3d SNIC model is a reduction of this more detailed model in which AMPA current is input into compartment 1, leak conductances are equally distributed throughout the 10 compartments, and the spike machine (sodium and delayed rectifier conductances) placed into compartment 10. The characteristic frequency of the noisy AMPA current pulses is 440 Hz (black). Fast negative feedback from low threshold voltage-gated potassium conductance spread throughout the 10 compartments extends the generator’s dynamic range to about 38 dB. The sample spike train is driven by an average of 1 vesicle released by the IHC per sound wave cycle and includes vesicle release noise, with some of the vesicles released by the simulated IHC having an incorrect phase. 1 mV of Gaussian voltage noise is included. For low noise the action potentials phase lock to the AMPA current pulses. By comparing the attenuation of the back propagating action potentials in compartment 1 (orange) to the generator’s action potentials in compartment 10 (blue) the model dendrite can be seen to have an electrical length of about 0.22 space constant for small input current. The dendrite “lengthens” to 0.26 space constant for large input by turning on more low threshold potassium conductance which increases its attenuation (a static potential is *e*-fold damped in 1 space constant). The epsps from compartment 1 (orange) are similarly attenuated after arriving at the generator compartment (blue).

Only Poisson noise in the number of vesicles released per sound wave cycle is included in the simple 3d SNIC model. Noise in the phase of IHC vesicle release and generator compartment noise such as Johnson noise are not included. Such noise is included in the biophysically-based 10 compartment model of the spike generator compartment where the action potentials mainly phase lock to the periodic AMPA current pulses, provided that these are larger than the typical noise currents within the generator compartment (for comparison a sample spike train shown in Fig. 4). This is likely why a single vesicle released from an IHC is made to cause such a large ∼40 pA current response in an ANF [10].

## Conclusions

We have shown how an ANF spike generator based on a 3d SNIC bifurcation is able to rate-code a wide input current range. Low threshold voltage-gated potassium conductance provides fast negative feedback that extends the generator’s dynamic range and linearizes its firing rate curve. Feedback from the lateral olive that turns off H current would be able to adjust the ANF’s dynamic range upwards to better encode a higher range of sound intensities. The olive’s likely purpose would be to increase the entropy of the spike trains that would typically occur within a higher noise environment in order to maximize the information content of the auditory encoding step. In a quiet environment, feedback from the olive that turns on H current would be able to make spike trains similar to those caused by synaptic input.

## Methods

Mathematica notebook with a simplified 3d model of the spike generator of a mammalian auditory nerve fiber. This 3d SNIC model is a reduction of a biophysically-based 10 compartment model of the ANF dendrite/spike generator. It contains only a fast voltage variable ***v***, slow high threshold potassium conductance gating variable ***n***, and a slower low threshold potassium conductance gating variable ***n_l_***. ANF input current is due to noisy periodic vesicle release from the IHC and here it is approximated by a Poisson random variable with an average of *no* vesicles released per sound wave cycle. Each vesicle release results in an increase of *dgA* in the AMPA conductance that charges the spike generator. The model can be run by cutting and pasting it into a Mathematica notebook.

(* Reduced ANF model based on a 3d SNIC bifurcation; units are dimensionless. Fast negative feedback from low voltage threshold potassium conductance linearizes its output and extends its dynamic range to about 48 dB. *).

f = 1.6; (*frequency of sound wave and AMPA current pulses*)

dgA = 0.20; (* size of AMPA conductance due to single vesicle release by IHC *)

eA = 0.0; (* reversal potential of AMPA conductance *)

no = 1.0; (* average number of vesicles released by IHC per sound wave cycle *)

Cm = 1.0; (* capacitance *)

gL = 5.0; (* amount of linear leak potassium conductance *)

gNa = 20.0; (* amount of fast voltage gated sodium conductance *)

eNa = 60.0; (* reversal potential of sodium conductance *)

mhalf = −20.0; (* half open voltage of m gate on instantaneously fast sodium conductance*)

msens = 15.0; (* voltage sensitivity of m gate on sodium conductance*)

gK = 10.0; (* amount of high voltage threshold potassium conductance *)

eK = −90.0; (* potassium conductance reversal potential *)

nhalf = −25.0; (*half open voltage of n gate on high voltage threshold potassium conductance *)

nsens = 5.0; (*voltage sensitivity of n gate on high threshold potassium conductance *)

tauK = 1.0; (* time constant for gating of n gate *)

gKl = 8.0; (* amount of low voltage threshold potassium conductance *)

nlhalf = −45.0; (*half open voltage of nl gate on low voltage threshold potassium conductance *)

nlsens = 5.0; (*voltage sensitivity of nl gate on low threshold potassium conductance *)

tauKl = 4.0; (* time constant for gating of nl gate *)

gH = 1.00; (* amount of H conductance *)

eH = −45.0; (* reversal potential of H conductance *)

(* Poisson clock vesicle release with average of *no* vesicles released per cycle *).

Ran = Table[RandomReal[],{i,1,1000}];

Table[PCVR[i] = 0.0,{i,1,1000}];

Do[Do[If[Ran[[i]] > Sum[Exp[-no] nôx/x!,{x,0,y}], PCVR[i] = PCVR[i]+1., 0.],

{y,0,50} ],{i,1,1000}];

(* differential equations *).

Eq1 = { Cm v′[t] -gA[t](v[t]-eA)-gH (v[t]-eH)-gL (v[t]-eK) -gNa minf[v](v[t]-eNa) -gKl nl[t] (v[t]-eK) - gK n[t](v[t]-eK)**}**;

Eq2 = {n′[t]  =  =  (ninf[v]-n[t])/tauK};

Eq3 = {nl′[t]  =  =  (nlinf[v]-nl[t])/tauKl};

(* auxiliary equations *).

minf[v_] : = 1./(1.+ Exp[(-v[t]+mhalf)/msens]);

ninf[v_] : = 1./(1.+ Exp[(-v[t]+nhalf)/nsens]);

nlinf[v_] : = 1./(1.+ Exp[(-v[t]+nlhalf)/nlsens]);

gA[t_]: = 0.0;

InitCond = {v[0] −50.0,n[0] 0.5,nl[0] 0.5};

Eqns =  Join[Eq1,Eq2,Eq3,InitCond];

(* initial NDSolve for half period without AMPA forcing *).

Subscript[ml,0] = NDSolve[Eqns,{v,n,nl}, {t,0.0,0.5/f}, AccuracyGoal→15];

(* 1000 more NDSolves, each for a half period with AMPA forcing *).

Do[{tstart  = 0.5/f * i; tstop  =  tstart +0.5/f;

InitCond = {v[tstart]Part[v[tstart]/. Subscript[ml,i−1],1],n[tstart] Part[n[tstart]/. Subscript[ml,i−1],1],nl[tstart] Part[nl[tstart]/. Subscript[ml,i−1],1]};

gA[t_]: =  PCVR[i] dgA Piecewise[{{Sin[2 Pi f t],Sin[2 Pi f t]>0.0},

{0.,!(Sin[2 Pi f t]>0.0 )}}];

Eqns =  Join[Eq1,Eq2,Eq3,InitCond];

Subscript[ml,i] = NDSolve[Eqns, {v,n,nl}, {t,tstart,tstop}, AccuracyGoal→15];

}, {i,1,1000}];

(* output plots *).

Print["no  =  ", no].

Print["gKl  =  ",gKl,"gL  =  ",gL," gH =  ", gH].

Do[{Subscript[vPlot, i] = Plot[v[t]/. Subscript[ml, i],{t, 0.5/f * i, 0.5/f * (i+1)},PlotRange->All,PlotStyle->PointSize[0.02]];}, {i,792,846}];

vp = Table[Subscript[vPlot, i], {i,792,846}];

Do[{Subscript[IinPlot, i] = Plot[−100+gA[t](v[t]-eA)/. Subscript[ml, i],{t, 0.5/f * i, 0.5/f * (i+1)},PlotRange->All,PlotStyle->{Black,PointSize[0.05]}];}, {i,792,846}];

Iinp = Table[Subscript[IinPlot, i], {i,792,846}];

curly = Show[vp, Iinp, AxesLabel->{"t", "v, I"}].

larry = Table[{First[v[i *0.5/f +j*0.005/f]/. Subscript[ml, i]],First[nl[i *0.5/f +j*0.005/f]/. Subscript[ml, i]],First[n[i *0.5/f +j*0.005/f]/. Subscript[ml, i]]}, {i,792,846}, {j,0,99}];

mo = ListPointPlot3D[larry,PlotStyle->Blue, AxesLabel->{"v", Style[Subscript[n, l],Bold],"n"}];

Show[mo, PlotRange->All].
